# Comparative analysis of porcine-uterine decellularization for bioactive-molecule preservation and DNA removal

**DOI:** 10.3389/fbioe.2024.1418034

**Published:** 2024-10-02

**Authors:** Abbas Fazel Anvari Yazdi, Kobra Tahermanesh, Maryam Ejlali, Amin Babaei-Ghazvini, Bishnu Acharya, Ildiko Badea, Daniel J. MacPhee, Xiongbiao Chen

**Affiliations:** ^1^ Division of Biomedical Engineering, University of Saskatchewan, Saskatoon, Canada; ^2^ Department of Obstetrics and Gynecology, School of Medicine, Iran University of Medical Sciences (IUMS), Tehran, Iran; ^3^ College of Pharmacy and Nutrition, University of Saskatchewan, Saskatoon, SK, Canada; ^4^ Department of Chemical and Biological Engineering, University of Saskatchewan, Saskatoon, SK, Canada; ^5^ Department of Veterinary Biomedical Sciences, Western College of Veterinary Medicine, University of Saskatchewan, Saskatoon, SK, Canada; ^6^ Department of Mechanical Engineering, University of Saskatchewan, Saskatoon, SK, Canada

**Keywords:** decellularization, extra-cellular matrix, uterus, porcine, tissue engineering, regeneration medicine

## Abstract

**Introduction:**

Decellularized uterine extracellular matrix has emerged as a pivotal focus in the realm of biomaterials, offering a promising source in uterine tissue regeneration, research on disease diagnosis and treatments, and ultimately uterine transplantation. In this study, we examined various protocols for decellularizing porcine uterine tissues, aimed to unravel the intricate dynamics of DNA removal, bioactive molecules preservation, and microstructural alterations.

**Methods:**

Porcine uterine tissues were treated with 6 different, yet rigorously selected and designed, protocols with sodium dodecyl sulfate (SDS), Triton^®^ X-100, peracetic acid + ethanol, and DNase I. After decellularization, we examined DNA quantification, histological staining (H&E and DAPI), glycosaminoglycans (GAG) assay, scanning electron microscopy (SEM), Fourier-transform infrared spectroscopy (FT-IR), X-ray photoelectron spectroscopy (XPS), and Thermogravimetric Analysis (TGA).

**Results:**

A comparative analysis among all 6 protocols was conducted with the results demonstrating that all protocols achieved decellularization; while 0.1% SDS + 1% Triton^®^ X-100, coupled with agitation, demonstrated the highest efficiency in DNA removal. Also, it was found that DNase I played a key role in enhancing the efficiency of the decellularization process by underscoring its significance in digesting cellular contents and eliminating cell debris by 99.79% (19.63 ± 3.92 ng/mg dry weight).

**Conclusions:**

Our findings enhance the nuanced understanding of DNA removal, GAG preservation, microstructural alteration, and protein decomposition in decellularized uterine extracellular matrix, while highlighting the importance of decellularization protocols designed for intended applications. This study along with our findings represents meaningful progress for advancing the field of uterine transplantation and related tissue engineering/regenerative medicine.

## 1 Introduction

Uterine transplantation has long been envisioned as a treatment for infertility in women affected by hysterectomy due to uterine cancer or those born with Mayer-Rokitansky-Küster-Hauser (MRKH) syndrome (Testa et al., 2022; Herlin et al., 2020). Owing to a shortage of uterine donors, alternative solutions have been exploring to address the long-term complications associated with infertility. The first live birth after uterus transplantation was reported in 2015, offering newfound hope for infertile women and turning a long-held dream into reality (Brännström et al., 2015). Similar to other organ transplantations, the scarcity of uterine donors and the necessity for long-term immunosuppression render this approach highly complex. While xenotransplantation has emerged to address the demand for organs and tissues, bioengineering of uterine organ from other species has also been explored as a more viable option to tackle the paucity of organ tissue donors (Sykes, 2022; Gullo et al., 2022).

Uterine complications arising from surgeries, such as cesarean sections and myomectomies, can also pose significant risks to both mother and fetus. These complications include morbidly adherent placenta, scar rupture or dehiscence, and cesarean scar ectopic pregnancy. In non-pregnant women, these surgeries can also lead to issues like dysmenorrhea, abnormal uterine bleeding, pelvic pain, dyspareunia, and subfertility (Tahermanesh et al., 2022; Tahermanesh et al., 2021). To address these complications, it is crucial to focus on strategies that regenerate damaged uterine tissue in a way that closely resembles and mimics the native extracellular matrix. This approach aims to effectively treat the scarred or wounded area (Hanuman et al., 2024; Hanuman et al., 2023).

Pre-clinical studies have identified that extracellular matrix (ECM) derived from reproductive tissue contains bioactive components and preserved tissue-specific microstructure (Francés-Herrero et al., 2023; Almeida et al., 2023). The utilization of uterine tissue-specific ECM could help to overcome the limitations of uterus transplantation.

ECM consists of essential components found in native tissue, including collagen and elastin as structural proteins, fibronectin and laminin as adhesion proteins, and various glycoproteins and proteoglycans as ground material (Kamaraj et al., 2022; McInnes et al., 2022; Chen et al., 2023). To obtain uterine-specific ECM, decellularization techniques have been developed over the last decades to recreate a natural microenvironment similar to native uterine tissue (Campo et al., 2016; Campo et al., 2019; Hellström et al., 2014; Arezoo et al., 2021; Daryabari et al., 2022). The preservation of these crucial proteins or bioactive molecules, and materials is achieved through efficient protocols, while removing the nuclear and intracellular components. Efficient decellularization not only reduces the risk of immune reaction, but also preserves more bioactive molecules (Sykes, 2022). Hence, the donor tissue source does not need to be the same as the host and can be collected from different species (Tiemann et al., 2020). The efficiency of uterine decellularization protocols has been continuously developed and optimized for a variety of species, including human (Daryabari et al., 2022; Sargazi et al., 2021), porcine (Campo et al., 2016; Ahn et al., 2023; Almeida et al., 2024), bovine (Sehic et al., 2024; Daryabari et al., 2019), cat (Sanguansook et al., 2024), sheep (Tiemann et al., 2020; Padma et al., 2021a; Dahm-Kähler et al., 2008), rat (Miyazaki and Maruyama, 2014; Charoensombut et al., 2022; Yoshimasa et al., 2023; Wang et al., 2023; Masoomikarimi et al., 2023; Padma et al., 2021b), and rabbit (Campo et al., 2019; Wang et al., 2023; Yao et al., 2020).

Generally, decellularization methods are divided into three categories of physical, chemical and enzymatic (Fernández-Pérez and Ahearne, 2019). The freeze-thawing technique is the most common physical decellularization, by which cell membrane is ruptured and thus removed (Rahman et al., 2018). However, this technique is not working effectively alone and needs complementary treatments, such as chemical detergents or enzymes, to wash and dissociate deoxyribonucleic acid (DNA) and other immunogenic components. Chemical treatments are the common methods for decellularization due to their disruptive effect on phospholipids and their ability to remove cell components. Among various chemical detergents, sodium dodecyl sulfate (SDS) is the most favored ionic detergent for decellularization, with a 90% removal rate of DNA in different species (Allu et al., 2023). However, it suffers with such drawbacks as cell toxicity and denaturation of proteins, sulfated glycosaminoglycans (GAGs), and growth factors (Kawasaki et al., 2015; Weng et al., 2021; Ott et al., 2008). It is suggested that SDS may not be an ideal detergent for decellularization, as it can cause the agglutination of DNA on the tissue surface in perfusion techniques (Meezan et al., 1975). Triton^®^ X-100, a non-ionic detergent, can selectively target lipid-lipid and lipid-protein interactions while preserving protein-protein interactions. It is predominantly used to generate matrices with a protein-dominant composition (Mendibil et al., 2020; Woods and Gratzer, 2005). While Triton^®^ X-100 may not be as effective as SDS in removing antigenic cell components, it exhibits a superior retention profile for bioactive molecules (Liu et al., 2018).

Peracetic acid (PAA) can lyse cells by disrupting phospholipid cell membranes (Nazari et al., 2012). PAA solubilizes cell membranes and nuclear components by ionic interactions (Gilpin and Yang, 2017). The efficiency of PAA depends on tissue thickness, i.e., it is less effective for intact tissues. In a comparative study PAA was the best method for preserving tissue structure and collagen fibers but was not sufficient to completely remove cellular components, with more than 80% of cell DNA fragments detected within the ECM (Poornejad et al., 2016).

Among enzymatic protocols, nucleases, such as deoxyribonuclease I (DNase I), are commonly used to break down nuclear and DNA components into smaller fragments, achieving a significant removal of cell components (Moffat et al., 2022). Enzymatic-assisted decellularization with DNase I has been successfully coupled with chemical decellularization to efficiently remove DNA fragments. In a study by Francés-Herrero et al., more than 99% of DNA components were removed by a combination of 0.1% SDS, 0.5% Triton^®^ X-100, and 60 U/mL DNase I (Francés-Herrero et al., 2023). Studies indicate that the addition of a DNase I wash step can also improve the retention of bioactive molecules (Wang et al., 2021). Tissues treated with DNase I must undergo extensive washing and numerous rinses to remove immunogenic compounds to prevent cell apoptosis after recellularization by host cells (Oliveri et al., 2001).

Though various methods or protocols for decellularization are available, it remains undiscovered with regard to their outcomes and optimization in decellularizing uterus for potential applications. As inspired, our study was aimed to examine the impact of six different, yet rigorously selected and designed, decellularization protocols, combining physical, chemical, and enzymatic techniques, on the production of decellularized uterine extracellular matrix (dUECM) derived from the porcine uterus. Notably, dUECM has found wide applications in uterine tissue engineering and regenerative medicine with varying degrees of success being associated with the decellularization protocols used. Our comparative analysis among these protocols examined in this paper will allow one to design and optimize the protocol for potential use in the subsequent studies in the field of uterine tissue engineering and regenerative medicine.

## 2 Materials and methods

### 2.1 Decellularization of porcine uterine

**FIGURE 1:**
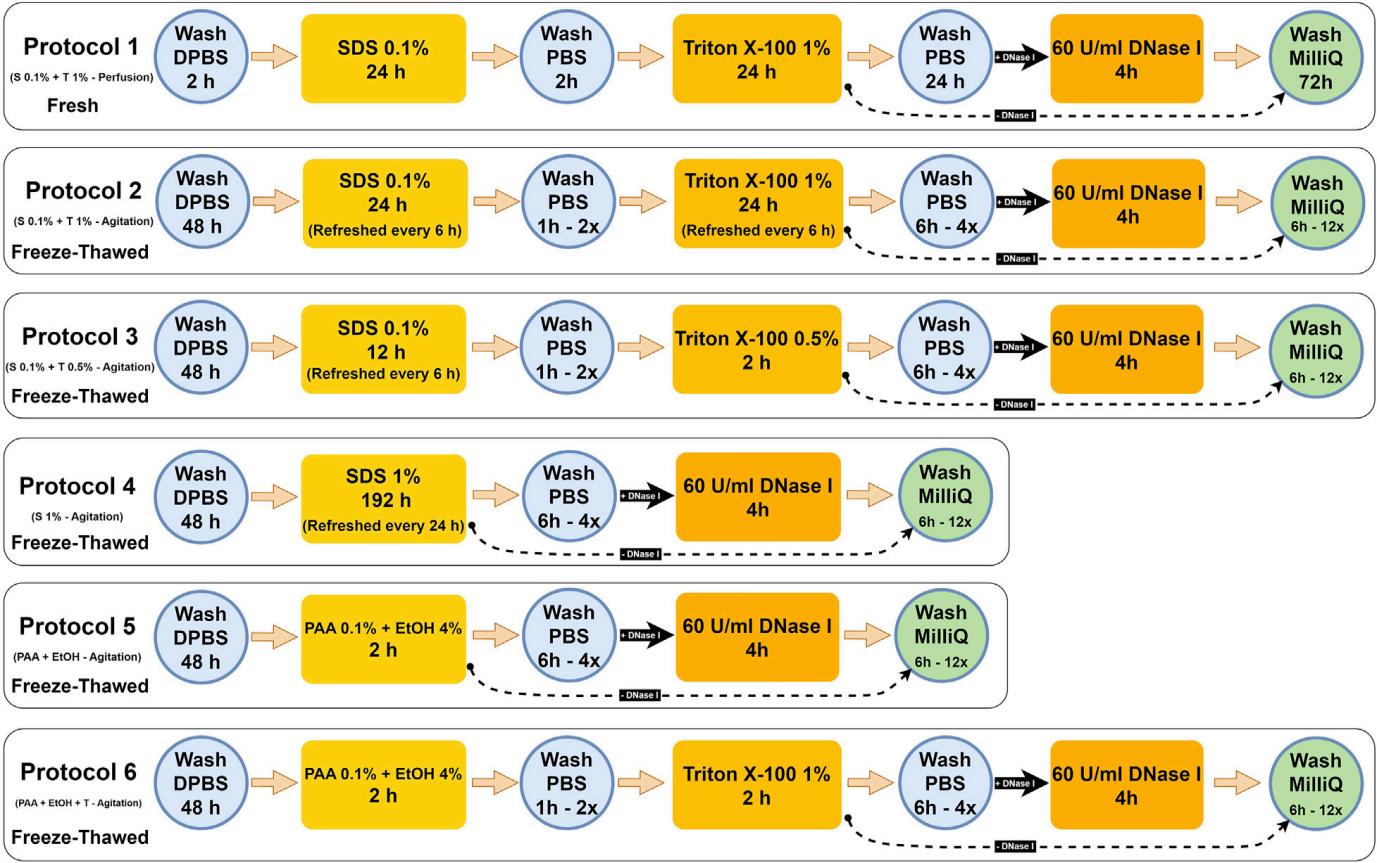
Detailed porcine uterine decellularization protocols. Hereinafter: Protocol 1 = S 0.1% + T 1% - Perfusion, Protocol 2 = S 0.1% + T 1% - Agitation, Protocol 3 = S 0.1% + T 0.5% - Agitation, Protocol 4 = S1% - Agitation, Protocol 5 = PAA + EtOH, and Protocol 6 = PAA + EtOH + T. The black straight arrow **(** → **)** indicates that the uterine tissues were treated with DNase I (+DNase I), and the black dashed arrow **(** − → **)** indicates that the tissues were not treated with DNase I (-DNase I).

Whole reproductive tissues were procured from 1-year-old pigs weighing approximately 200 kg (*n = 6)* from a local slaughterhouse (Friesen’s Meat Processing Inc., Warman, Saskatchewan, Canada). The tissues were immediately submerged in 1x sterile phosphate buffered saline (PBS; Sigma-Aldrich, Cat.#P4417, United States) and transported within 30 min to the lab for immediate processing. For Protocol 1, adopted from Campo et al. (Campo et al., 2016), the whole uterine organ, including the broad ligaments, veins, and arteries, was preserved for canulation. Briefly, after the organ was canulated with 20-G cannulas, the arteries were washed and dilated with PBS and then infused by a peristaltic perfusion pump (Cole-Parmer Instruments, Cat.# 77200-60, United States) using L/S 17 tubing (Masterflex^®^ L/S 17, Cat.# 060508-17) at a flow rate of 15 mL/min. A detailed protocol is given in Figure 1.

For protocols 2–6, the ovaries, fallopian tubes, broad ligaments, and cervix were dissected, and the uterine tissues were preserved in a −80°C freezer for subsequent use. For initiating the decellularization, the frozen tissues were thawed overnight in the fridge and transversally sliced into ∼2 mm discs, which were then washed with sterilized Dulbecco’s phosphate-buffered saline (DPBS pH 7.4; Gibco, Cat.# 21600, United States) with 1% antibiotic/antimycotic solution (Anti-Anti; Gibco, Cat.# 15240062, United States). Each set of sliced tissues (30 gr/500 mL DPBS) was transferred into 1,000 mL Erlenmeyer flasks and placed on an orbital shaker. The tissues were continuously agitated at 200 rpm for 48 h at 4°C to remove any remaining blood residues thoroughly. In the first 24 h, the DPBS solution was replaced three times at 8-h intervals. The uterine tissues were then sequentially treated with different types of reagents, including SDS (Sigma-Aldrich, Cat.#L3771, Japan), Triton^®^ X-100 (Triton; Fisher Bioreagents, Cat.# BP151, Canada), PAA (Sigma-Aldrich, Cat.# 269336, United States), anhydrous ethyl alcohol (EtOH; Commercial Alcohol, Canada) and DNase I (Worthington Biochemical, Cat.# LS002145, United States). The tissues were washed with ultrapure deionized H_2_O (MilliQ) by aspirating the media between each treatment to remove the previous reagent and cellular debris. In total, six decellularized treatments were compared (Figure 1).

**FIGURE 2:**
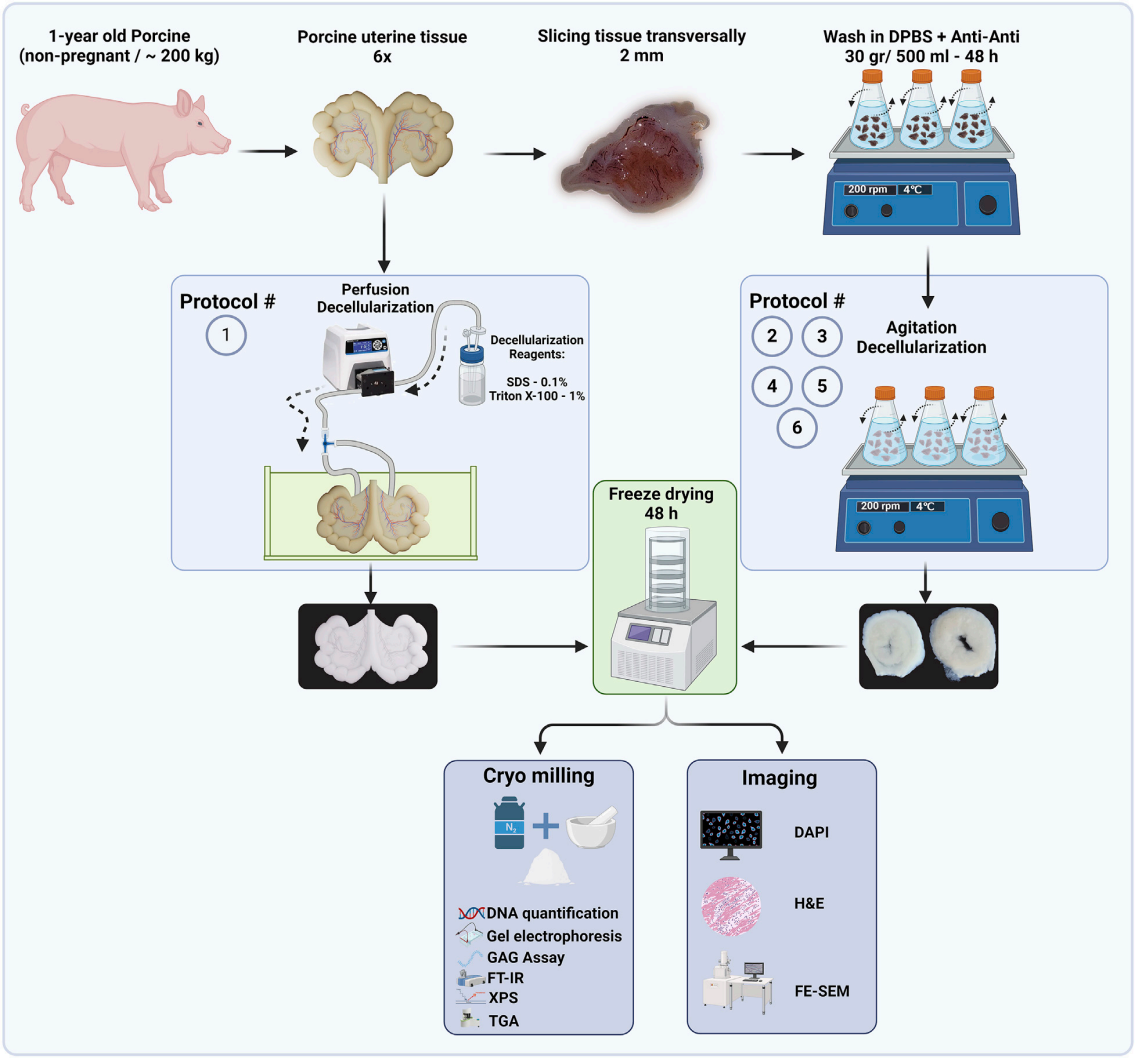
Flow diagram of decellularized protocols and proposed techniques to quantify the efficiency of decellularization procedures (Created with BioRender.com).

In the next step, the tissues for each protocol were freeze-dried (LACONCO, United States) at −50°C under 0.05 mBar pressure for 48 h. Subsequently, the freeze-dried tissues were crushed into fine ECM powders (∼400 µm) using liquid nitrogen and a mortar and pestle. These powders were used in subsequent quantification (Figure 2).

### 2.2 Microstructural analysis

#### 2.2.1 DNA quantification and fragment-size determination

To quantify the remaining DNA content in decellularized tissues, 10 mg dry tissue was used in triplicates for each protocol. DNA content was extracted using a commercial kit (DNeasy^®^ Blood and Tissue Kit; Qiagen, Cat# 69504, Germany) according to the manufacturer’s instructions, and DNA yield (ng/mg) was quantified spectrophotometrically (γ = 260 nm) by Nanodrop^®^ Spectrophotometer (Thermo Scientific 2000c, United States). To visualize the DNA fragments, samples were loaded with DNA Gel Loading Dye (Thermo Scientific, R0611, United States) and assessed on a 2% agarose gel containing SYBR Safe (Invitrogen, Cat.#S33102, United States) for a total runtime of 90 min (60 min at 75 V and 30 min at 90 V). As a reference, 1 kb plus DNA ladder (Invitrogen™, Cat.# 10787018, United States) was used for comparison. Multiple exposure times were acquired using a Bio-Rad ChemiDoc™ MP digital imaging system to visualize the DNA smears.

#### 2.2.2 GAGs assay

GAG content of the decellularized tissues was determined by digesting triplicates of 100 mg freeze-dried samples in 20 μL/mL proteinase K (Qiagen, Cat.# 19131, pH 8, Germany) and incubation overnight at 56°C. Enzyme inactivation was performed at 90°C for 10 min. GAG concentrations were calculated at pH 1.5 using the 1,9-dimethyl-methylene blue (DMMB; Sigma, Cat.# 341088, United States) assay (Zheng and Levenston, 2015). Briefly, 20 μL of extracted GAG was added to 200 μL of DMMB dye, and the optical density (OD) was measured immediately with a Plate Reader Spectrophotometer (Molecular Devices SpectraMax 190, United States) at 650 nm was used (*n = 3*). A standard curve was constructed using heparin (Sigma-Aldrich, Cat.#H3393-250KU, United States).

#### 2.2.3 H&E and DAPI staining

Native and decellularized tissues (Protocols 1-6) were fixed in 10% neutral buffered formalin (NBF; Sigma-Aldrich, Cat# HT501128, United States) for 24 h at ambient temperature, embedded in paraffin, and sectioned at 10 μm thickness. To validate cell removal, the sections were stained with hematoxylin and eosin (H&E) and 4′,6-diamidino-2-phenylindole (DAPI; Sigma, Cat# MBD0015, Israel). Slides stained with DAPI were treated with ProLong™ Diamond Anti-Fade Reagent (ThermoFisher, Cat #P36931), covered with micro cover glass (VWR, Cat.# 48404-454, United States) and imaged with an automated microscope (BioTek Lionheart LX, United States).

#### 2.2.4 Scanning electron microscopy (SEM)

The microstructure of the decellularized tissues was investigated using field-emission scanning electron microscopy (FE-SEM; Hitachi SU8010, Japan). Preparation of both native and decellularized tissues followed previously described protocols (Herlin et al., 2020; Brännström et al., 2015; Sykes, 2022). Samples were fixed using 2.5% glutaraldehyde (GA; Polysciences Inc., Cat.# 00376, Germany) at 4 °C overnight, then gradually dehydrated via an increasing concentrations of EtOH (10%, 30%, 60%, 90%, and 100%) (Mehdizadeh et al., 2014; Mehdizadehkashi et al., 2017). After dehydration, the samples were immersed into 1:2 and 2:1 hexamethyldisilizane (HMDS; Thermo Scientific, Cat.# A15139. AP, Germany): absolute EtOH, respectively, for 20 min and then 100% HMDS solution overnight. Samples were air-dried in a fume hood. Specimens were mounted on aluminum stubs with double-sided carbon tape and coated with 10 nm of gold (Quorum Q150TES Sputter Coater) as the conductive layer. For imaging, the voltage was fixed at an accelerating voltage of 3 KV to capture the secondary electron mode images to prevent damage to the tissue surface from electron bombardment. The ImageJ 1.54g software was employed for the measurement of ECM fibers (Schneider et al., 2012).

#### 2.2.5 FT-IR spectroscopy

IR spectra of the freeze-dried native and decellularized specimens were acquired using Attenuated Total Reflectance-Fourier Transform Infrared spectroscopy (ATR-FTIR; Spectrum 3 Tri-Range MIR/NIR/FIR Spectrometer, PerkinElmer, United States). The decellularized specimens were placed directly on the ATR crystal (*n = 3*). FT-IR spectra were acquired in 4,000–600 cm^−1^ range at a resolution of 4 cm^−1^.

#### 2.2.6 X-ray photoelectron spectroscopy (XPS)

The XPS analysis was performed at the Saskatchewan Structural Sciences Centre (SSSC) employing an AXIS Supra system from Kratos (Manchester, United Kingdom) to collect the X-ray Photoelectron Spectroscopy (XPS) data. The system uses a 500 mm Rowland circle monochromated Al K-α (1,486.6 eV) source and a combined hemispherical and spherical mirror analyzer (HSA/SMA). A spot size of hybrid slot (300 × 700) microns was used. In the survey scan of native and decellularized samples (*N = 3*), binding energies of 0–1,200 eV were collected in steps of 1 eV with a pass energy of 160 eV. The high-resolution scan was collected in 0.1 eV steps. To quantify the differences in carbon content among the samples, we employed the peak area method for comparison of the full width at half maximum (FWHM) of the high-resolution peaks. We chose C1 (C-C/C-H) as the internal standard for this analysis due to its relatively higher bond stability in comparison to other carbon types (C2 and C3), which are less prone to significant alterations. CASA XPS was used to analyze the data (Fairley et al., 2021).

#### 2.2.7 Thermogravimetric analysis (TGA)

To assess the protein content in each decellularization protocol, thermogravimetric analysis (TGA) was performed using a Perkin-Elmer TGA 8000 analyzer. Approximately 5 mg of the freeze-dried native and decellularized samples underwent heating from 50°C to 600°C at a rate of 10°C/min under a constant nitrogen gas flow of 30 cm^3^/min. By differentiating the TGA values, the differential form of TGA (DTG) was derived, facilitating the identification of the maximum disintegration temperature at each stage of thermal degradation.

#### 2.2.8 Statistical analysis

Data were analyzed using SPSS software version 27.0 (SPSS Inc., Chicago, IL, United States) with the quantitative variables reported as mean ± SD. The normality of data distribution was assessed using the Shapiro-Wilk test, and appropriate statistical tests were selected based on the results. A two-way analysis of variance (ANOVA) was conducted to evaluate differences in DNA content among groups, considering both main effects and interactions involving DNase I treatment. For post-hoc pairwise comparisons, Dunnett’s test was employed to compare each experimental group with the designated control group. One-way ANOVA was used to compare the distribution of GAG content between protocols with post-hoc Dunnett’s test to compare each protocol to the control group. Due to the non-normal distribution of FT-IR spectral absorption, differences between groups were evaluated by using the Kruskal-Wallis test, followed by Dunn’s test for multiple non-parametric comparisons. MagicPlot 3.0.1 was used for curve fitting and area measurement in TGA and FT-IR experiments. Graphs were created using GraphPad Prism software (GraphPad Software Inc., San Diego, CA, United States). The significance levels are indicated as follows: **** for *p* < 0.0001, *** for *p* < 0.001, ** for *p* < 0.01, * for *p* < 0.05, and NS for non-significant differences.

## 3 Results and discussion

There is no standard for decellularization efficiency; to achieve the optimal decellularization, the following criteria are proposed: a) the remaining DNA should be less than 50 ng/mg in dried condition, b) the DNA fragments to be less than 200 bp and c) no visible nucleic material in decellularized tissues (McInnes et al., 2022; Crapo et al., 2011; Zheng et al., 2005).

### 3.1 DNA quantification, GAGs assay, fragment size determination and histological assessments

**FIGURE 3:**
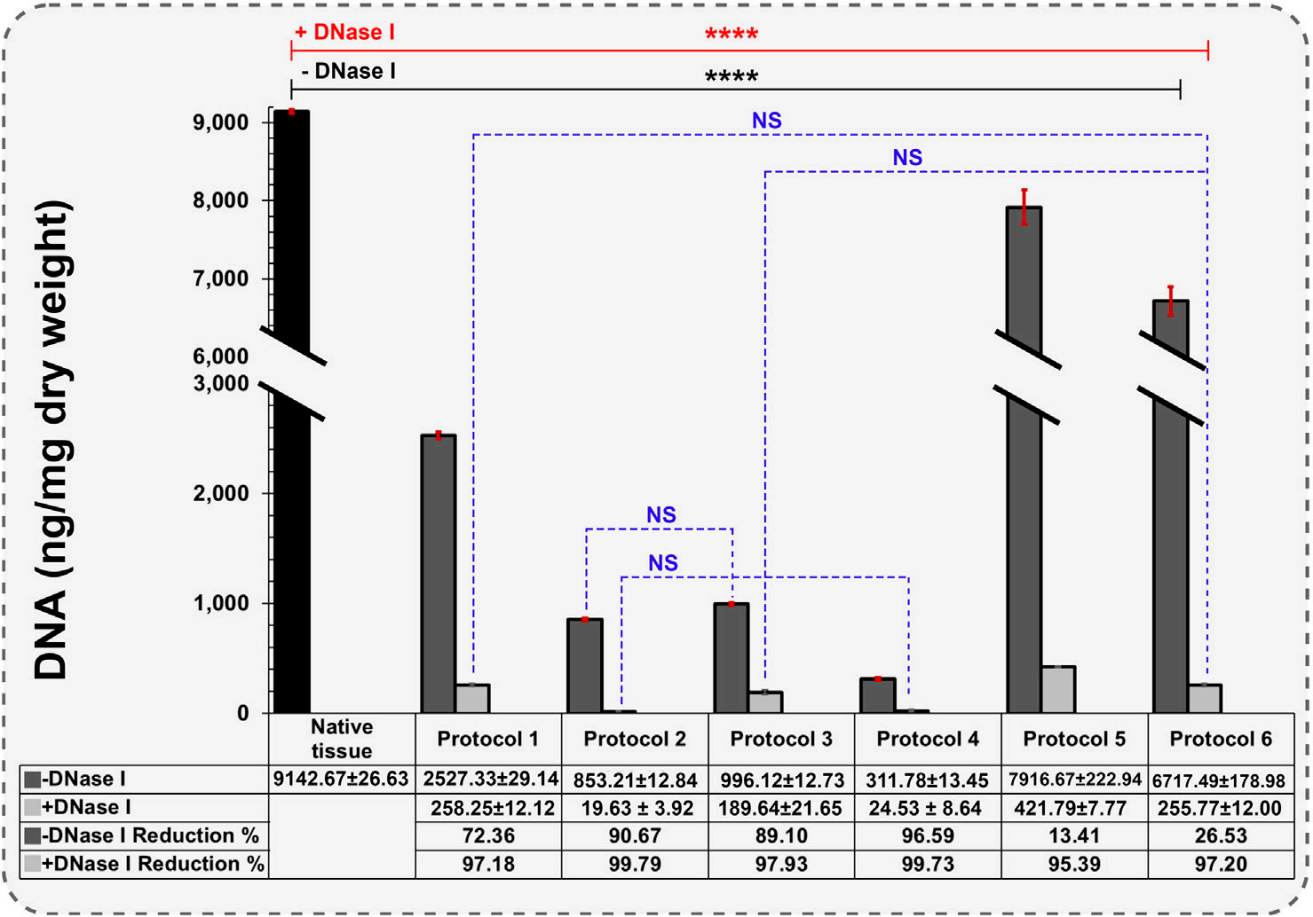
Quantification of remaining DNA components in different protocols (Data are shown as M±SD). -DNase I: Without DNase I treatment, +DNase I: With DNase I treatment. A two-way analysis of variance was performed to evaluate differences in DNA content among groups, for post-hoc pairwise comparisons, Dunnett’s test was used to compare each experimental group with a designated control group.

Throughout the decellularization procedures, the color of uterine tissues underwent visual change from red to white, resulting in a semi-transparent appearance, indicative of the removal of blood and cells. The initial freezing and thawing process in Protocols 2 - 6 induced cellular lysis, leading to the rupture of membranes due to water crystal growth (Liu et al., 2019). DNA content in native tissue was 9142.66 ± 25.63 ng/mg dry weight and significantly reduced after each decellularization protocol (Figure 3). Protocols 2 and 4 exhibited the highest efficiency for DNA removal, with the remaining DNA smears without DNase I treatment measured at 853.21 ± 12.84 and 311.78 ± 13.45 ng/mg dry weight, respectively, higher than the threshold of 50 ng/dsDNA per mg dUECM dry weight. After DNase I treatment, the measured values decreased to 19.63 ± 3.92 and 24.53 ± 8.64 ng/mg dry weight, respectively, representing a 99.79% and 99.73% reduction compared to the native tissue (*N* = 3, *p* = 0.0001). Protocols 1 and 3 were able to significantly remove DNA content (97.18% and 97.93% reduction, respectively), and thus showed higher efficiency than protocols 5 and 6 (95.39% and 97.20% reduction, respectively). Notably, our findings indicate that Protocol 2 (0.1% SDS and 1% Triton^®^ X-100) using an agitation technique was more effective in terms of DNA removal than the perfusion protocol.

In a similar study by Campo et al., DNA quantification was performed on wet tissues rather than freeze-dried samples. The quantified DNA in native tissue was 1571.41 ± 427.17 ng/mg wet tissue. Upon applying the perfusion technique to Freeze-Thawed and fresh uterine tissue, they observed 139.604 ± 89.561 and 40.792 ± 16.462 ng/mg wet tissue, respectively, representing 90.94% and 97.36% DNA reduction compared to the native uterus (Campo et al., 2016). A limitation of this study is the use of wet tissue for DNA quantification. Using wet tissue results in capturing less DNA for each test than under dry conditions, and it may not represent the actual condition of our desired tissue accurately. According to studies, porcine uterine tissue has one of the highest moisture contents at 82.71%, and freeze-drying the tissue before DNA quantification is necessary to obtain promising data (Seong et al., 2014).

The levels of GAG preservation across different decellularization protocols significantly impact the mechanical properties and bioactivity of the decellularized uterine matrix. GAGs are integral to the extracellular matrix (ECM), contributing to its structural integrity by interacting with collagen fibers, thereby enhancing tissue resistance to compressive forces and overall mechanical strength (Menezes et al., 2023; Katta et al., 2008; Ghadie et al., 2023). ECM with better-preserved GAGs maintain the matrix’s natural biomechanical properties better, which is important to subsequent implantation and functional tissue regeneration. Moreover, GAGs, such as hyaluronic acid and chondroitin sulfate, are essential for tissue elasticity and flexibility, which are vital for tissues subjected to dynamic mechanical stress (Dovedytis et al., 2020; Mihajlovic et al., 2022).

In terms of bioactivity, GAGs are involved in cell signaling pathways that regulate cellular behaviors, including proliferation and differentiation (Silva et al., 2019; Wang et al., 2017). The retention of GAGs in the decellularized matrix ensures the preservation of these bioactive signals, facilitating cell attachment and growth (Papy-Garcia and Albanese, 2017). Additionally, GAGs are able to bind growth factors and other bioactive molecules so as to concentrate them within the ECM and thus to enhance the matrix’s regenerative potential (Kjellén and Lindahl, 2018). Also, it is noted that some GAGs have immunomodulatory effects on the host’s immune response, potentially reducing inflammation and promoting better tissue integration. Thus, preserving GAGs is crucial for maintaining the structural and functional integrity of the ECM, making it more suitable for tissue engineering applications (Yang et al., 2024).

**FIGURE 4:**
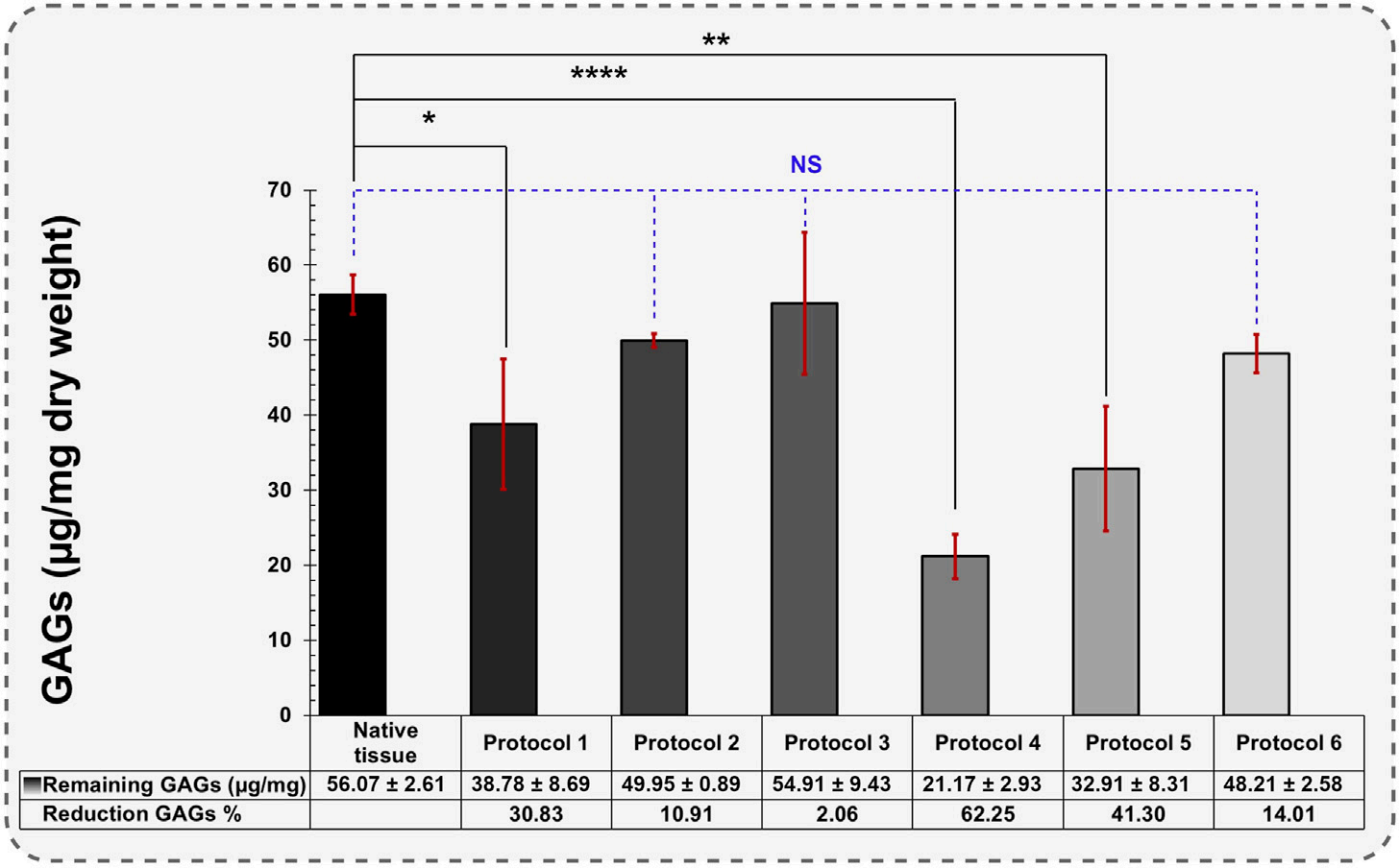
Quantification of remaining GAGs in different protocols (Data are shown as M±SD). A one-way analysis of variance (ANOVA) was performed to evaluate the quantification of remaining GAGs in different protocols. For post-hoc pairwise comparisons, Dunnett’s test was used to compare each experimental group with a designated control group.

In GAGs assay, the native tissue contained 56.07 ± 2.61 μg/mg of dry-weight GAGs (Figure 4). The levels of GAGs in Protocols 2 and 3 were maintained after decellularization with 0.1% SDS + 0.5 and 1% Triton^®^ X-100 reagents, measuring 49.95 ± 0.89 and 54.91 ± 9.43 μg/mg of dry weight, respectively (*p > 0.05*). In Protocol 1, the measured GAGs were 38.78 ± 8.69 μg/mg, representing a 30.83% reduction compared to native tissue. This significant reduction demonstrates that the perfusion technique removed more GAGs than the agitation technique (10.91% and 2.06% reduction, respectively). The greatest GAGs loss occurred using Protocol 4 after 192 h of 1% SDS treatment under immersed and agitated conditions (21.17 ± 2.93 μg/mg). GAGs loss experienced the highest level of reduction due to prolonged exposure to 1% SDS, reducing the GAGs components to 62.25% (*p < 0.0001*).

The reduction in GAGs occurring with different SDS treatments is consistent with findings in previous studies (Sykes, 2022; Gullo et al., 2022; Tahermanesh et al., 2022). Protocol 3 retained the highest levels of GAGs, likely due to its shorter exposure to 0.1% SDS (12 h). Using Triton^®^ X-100 in 0.5% concentration did not affect GAGs decomposition, demonstrating Triton^®^ X-100 ability to preserve GAGs within a specific timeframe.

In Protocols 5 and 6, the remaining GAGs also decreased significantly (32.91 ± 8.31 and 48.21 ± 2.58 μg/mg, respectively). These results suggest that PAA drastically reduces GAG components. This demonstrates that Triton^®^ X-100 performs better than PAA in preserving GAGs during the decellularization.

**FIGURE 5:**
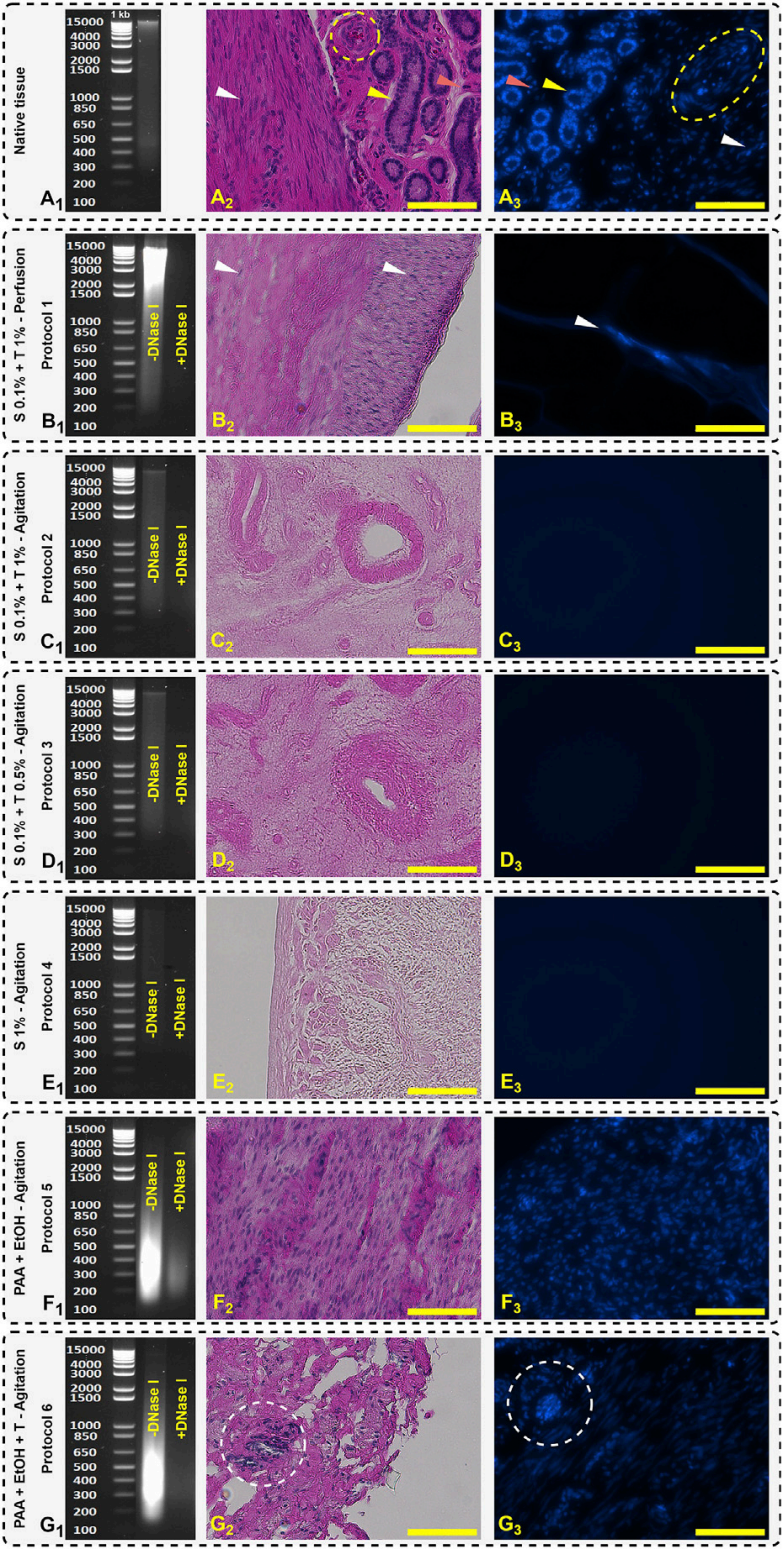
Agarose gel electrophoresis separation of DNA, H&E, and DAPI staining. Native tissue **(A)** represents the genomic DNA ladder fragments **(A_1_)**, glandular (yellow arrow), stromal cell (red arrow), vascular (yellow dashed circle in H&E and oval in DAPI), and smooth muscle cells (white arrow) **(A_2_, A_3_)**. Protocol 1 **(B)** shows broad DNA smears and some detectable nuclei in H&E and DAPI staining **(B_2_, B_3_)**. Protocols 2 **(C_1_, C_2_, C_3_)**, 3 **(D_1_, D_2_, D_3_)** and 4 **(E_1_, E_2_, E_3_)** show the absence of nuclear components which is representative of tissue decellularization and retention of the structure of ECM. Protocols 5 **(F_1_, F_2_, F_3_)** and 6 **(G_1_, G_2_, G_3_)** depict a less efficient decellularization indicated by visible nuclei (white dashed circle). Scale bars: 100 µm.

Decellularization efficiency of the different protocols, determined by genomic DNA measurements, H&E and DAPI staining for native and treated tissue are shown in Figure 5. Both H&E and DAPI staining indicated that in Protocols 2, 3 and 4, the tissues appeared to be devoid of DNA contents, however, quantitative analysis by electrophoresis still showed some DNA smearing, which was insignificant compared to other protocols (Figures 5C_1_, D_1_, E_1_). Residual DNA fragments in all protocols after DNase I treatment were less than 200 bp except for protocol 5 (Figure 5F_1_). Agarose gel electrophoresis analysis also confirmed that after DNase I treatment, DNA bands were not detectable.

In Protocol 1, the DNA smear mostly accumulated in the range of 1,500–15,000 bp which indicates that the decellularization protocol destroyed the cell membrane, but it was not efficient to remove and wash out all the cellular fragments. Due to the closed system in perfusion technique, the DNA remained trapped inside the ECM resulting in a high level of DNA content being detected. In H&E and DAPI images, the detergents were unable to diffuse into the intact areas and the cell nuclei observed indicated by white arrows (Figures 5B_2_, B_3_).

In accordance with the observations made by Campo et al. the decellularization of fresh porcine uterine tissue exhibited a higher efficiency when compared to tissue subjected to the freeze-thaw method (Campo et al., 2016). This was achieved through the utilization of the SDS and Triton^®^ X-100 perfusion technique. Residual DNA following the freeze-thaw and fresh protocols yielded values of 139.604 ± 89.561 and 40.792 ± 16.462 ng/mg wet tissue, respectively (Campo et al., 2016). In a follow-up study on rabbit uteri decellularization, a modified protocol with DNase I treatment was used and tissue washed with DNase I solution (2 μg/mL) for 1 h and effectively reduced the remained DNA to 10 ± 4 ng DNA per mg wet tissue (Campo et al., 2019). Thus, in our study, to achieve equivalent levels of DNA, we used SDS and Triton^®^ X-100 surfactant concentrations of 0.1% and 1%, respectively. The required incubation time for each was 24 h.

In Miyazaki et al. study, they applied perfusion technique with graded SDS perfusion (0.01, 0.1, and 1% wt./vol - 24 h for each wash) on rat uteri and subsequently treated with 1% Triton^®^ X-100 for 30 min (Miyazaki and Maruyama, 2014). After implantation of the decellularized tissue, recellularization occurred and the rats became pregnant 28 days after transplantation with success rate of 75%. Rat uteri is significantly smaller than the mature pig uteri and the concentration of detergents and time dependency is crucial to achieve efficient decellularization.

In PAA decellularization (Protocol 5 and 6), due to the high oxidizing effect of PAA, the cell membrane was effectively destroyed (Figures 5F_1_, G_1_), but it was not effective to remove and wash all DNA fragments with the agitation technique. As shown in Figure 5F_1_ (Protocol 5) and Figure 5G_1_ (Protocol 6), PAA and EtOH could destroy the DNA to smaller fragments and accumulated between 200–650 bp. In Protocol 6, after treatment with 1% Triton^®^ X-100, the DNA content decreased significantly compared to Protocol 5 (255.77 ± 12.00 and 421.79 ± 7.77 ng/mg dry weight, respectively), but higher than other protocols treated with the combination of SDS + Triton^®^ X-100 reagents. In histological studies, Protocols 5 and six show distinct nuclei in H&E and DAPI staining (Figures 5F_2_, F_3_, G_2_, G_3_, yellow dashed circle). Syed et al. reported that after PAA treatment, the tissue stiffness increased and decellularization efficiency was time dependent (Syed et al., 2014). For intact tissues like uterus, during the PAA and EtOH treatment for protocols 5 and 6, an increase in tissue stiffness was visually observed in our study. Syed et al. also reported that the decellularization with PAA after 2 h was not significantly effective compared to control group and the number of histologically visible nuclei was similar (Syed et al., 2014). In our findings, this behavior existed, and a large number of visible nuclei were visible. The efficiency of PAA depends on the duration and techniques of decellularization, as well as the tissue’s shape. Hence, the agitation technique for 2–3 mm sliced tissues, along with 2 h of PAA and EtOH treatment, followed by an additional 2-h post-treatment with Triton^®^ X-100, is not effective for pig uterine decellularization (Figure 5A_2_ compared to Figures 5F_2_, G_2_).

### 3.2 Ultrastructural study of dUECM

**FIGURE 6:**
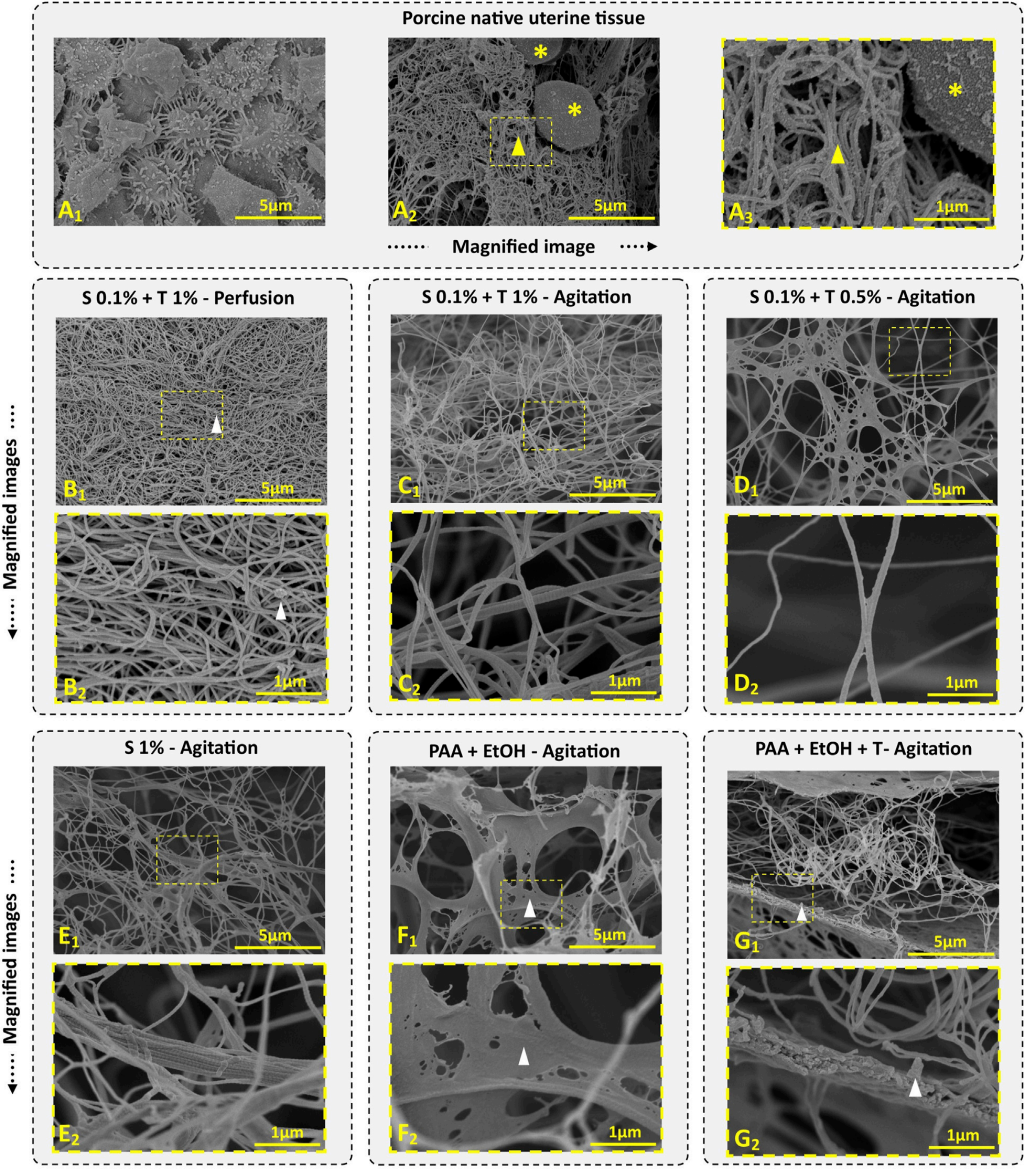
Porcine uterine tissue and dUECM SEM images. Porcine endometrial tissue comprises an epithelial layer and microvilli **(A_1_)**, along with ECM (indicated by yellow arrow) and stromal cells (indicated by yellow asterisks) **(A_2_, A_3_)**. In Protocol 1, we observed well-integrated ECM with some residual cellular content (depicted with white arrow) **(B_1_, B_2_)**. Protocol 2 reveals irregularly interlocked collagen fibers due to the agitation process **(C_1_, C_2_)**. In Protocol 3, a short treatment of 12 h with 0.1% SDS + 0.5% Triton^®^ X-100 successfully removes cellular contents while preserving more ECM components (D_1_, D_2_). Protocol 4, the longest and most aggressive decellularization treatment, results in the persistence of large collagen fibers, with collagen fibers interlocking irregularly after 192 h of agitation **(E_1_, E_2_)**. Protocols 5 **(F_1_, F_2_)** and 6 **(G_1_, G_2_)** display higher ECM density and residual cellular contents (depicted within the white arrow).

Ultrastructural properties of decellularized uterine tissues were examined using SEM. This SEM study focused on analyzing the retention of ECM. Collagen fibers exhibit a remarkable self-assembly capability, spontaneously organizing themselves into D-periodic cross-striated triple-helix fibrils. These fibrils elongate continuously, typically spanning a length of approximately 300 nm (Nashchekina et al., 2020). The native uterine endometrial tissue displayed epithelial cells and microvilli (Figure 6A_1_). The cross-sectional image of native tissue (Figures 6A_2_, A_3_) revealed the presence of stromal cells (marked by asterisk) and intact connective tissue (marked by a yellow arrow), displaying cellular structures indicating tissue integrity. In contrast, none of the decellularization protocols showed evidence of intact smooth muscle, stromal or epithelial cells.

In Protocol 1, the SEM image indicated fiber entanglement and dense ECM retention (Figure 6B_1_), however, small cell fragments were observed in some areas (Figure 6B_2_, white arrow). The collagen fiber thickness for the perfusion technique measured 53.45 ± 9.09 nm, which was thinner than that of native tissue (81.89 ± 21.17 nm).

In Protocol 2, ECM density and integration were lower compared to Protocol 1, but no cellular contents were detected (Figure 6C_1_). The fibers in this tissue exhibited collagen fibers, signifying retention of the ECM structure (Figure 6C_2_). The fiber thickness in this group was 61.02 ± 14.88 nm, which was thicker than Protocol 1.

Protocol 3 showed ECM integrity and the presence of fiber networks and connective tissues (Figures 6D_1_, D_2_). The fiber diameter in this group was 65.10 ± 13.68 nm.

In Protocol 4, the SEM images revealed complex collagen fibers, demonstrating an irregular network of collagen fibers due to prolonged agitation. As shown in Figure 6E_1_, E_2_, agitation for 192 h disrupted the networks, resulting in large collagen fibers (83.98 ± 22.97 nm) with randomly interlocked collagen fibers.

For Protocol 5 and 6, SEM micrographs displayed an intact microstructure with no noticeable differences at the ultrastructural level compared to native tissue (Figures 6, F_1_, F_2_, G_1_, G_2_). These two groups exhibited very thick and porous lamellar structures containing cell debris. In the PAA and EtOH treatment (Protocol 5), collagen fibers were less obvious, but after Triton^®^ X-100 treatment (Protocol 6), more collagen fibrils became visible. Both protocols demonstrated well-organized porosity, ensuring continuous retention of ECM protein fibers. The average protein fiber thickness was 85.07 ± 27.33 nm for Protocol 5 and 84.99 ± 19.71 nm for Protocol 6.

It’s worth noting that differentiating between collagen types or other proteins through SEM characterization is impossible. Various types, including types I, II, III, V, and IX, known as fibril-forming collagens, align into giant fibrils. Type IV creates an interlacing network in basement membranes, while type VI forms microfibrils, and type VII forms anchoring fibrils (Amirrah et al., 2022).

Similarly to this work, multiple studies have confirmed that decellularization with SDS and Triton^®^ X-100 effectively removes most cellular components while preserving ECM proteins (Francés-Herrero et al., 2023; Campo et al., 2016; Campo et al., 2019; Miyazaki and Maruyama, 2014).

### 3.3 Characterization of functional groups of the dUECM

**FIGURE 7:**
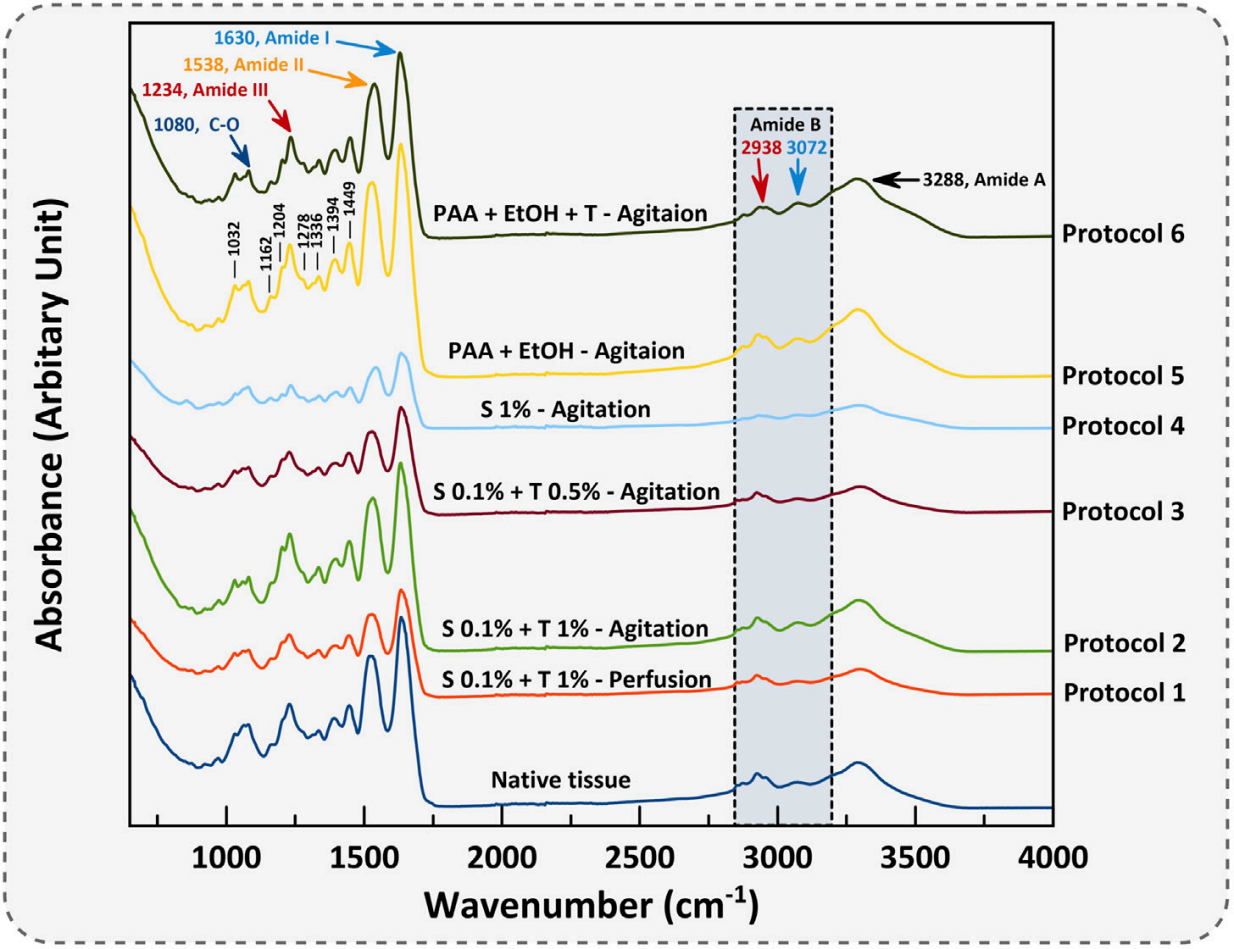
The mean FT-IR spectra of porcine uterine tissue and dUECM are presented. The IR spectrum classifies functional groups for each protocol.

**FIGURE 8:**
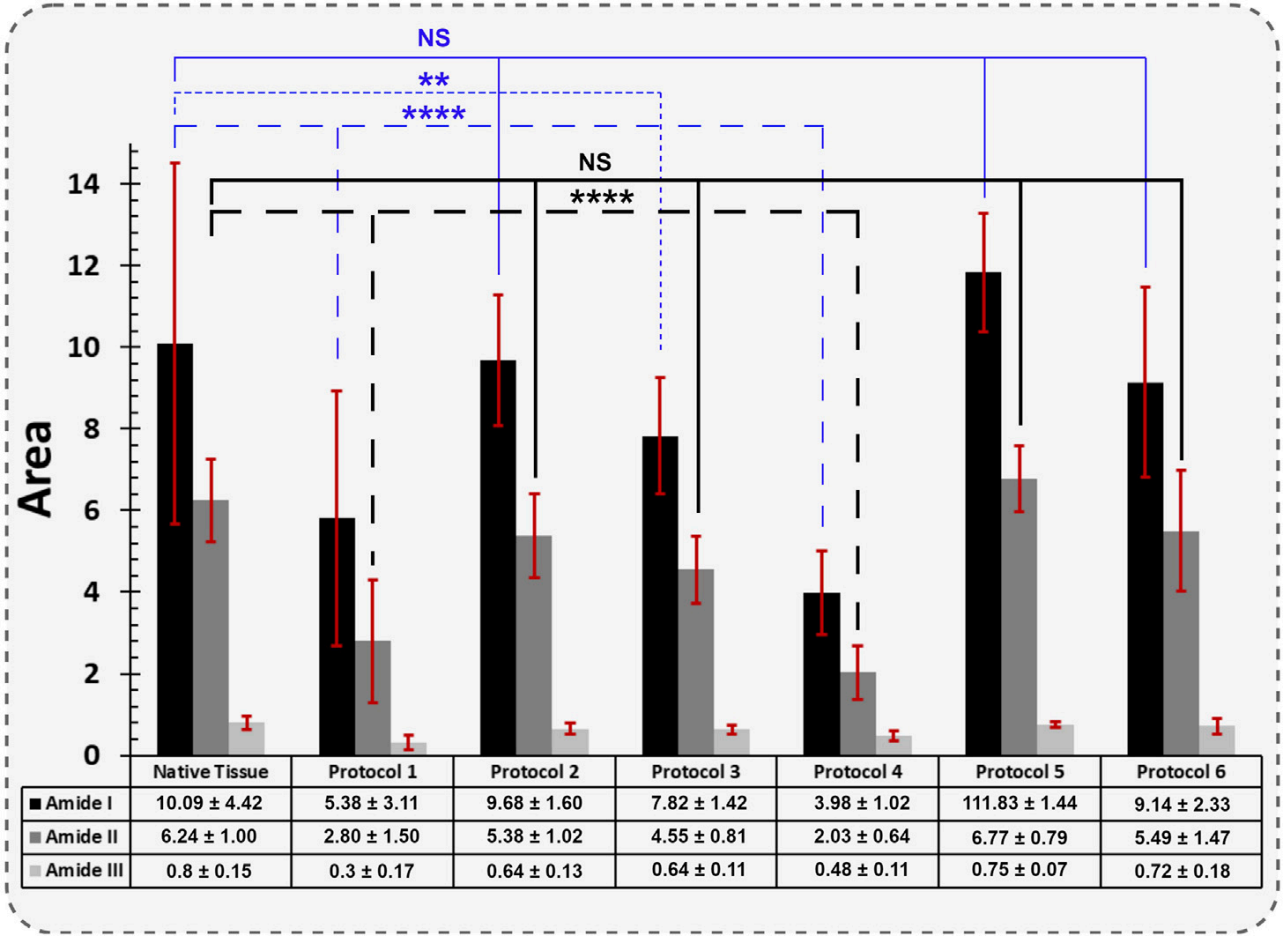
The quantified FT-IR spectral absorption area of the amide I, II, and III bands are shown. The profiles were processed for peak fitting to obtain the main amide components’ peak area. The values obtained for both Protocol 1 and 4 depicted the most significant difference for the Amide I and II functional groups compared to native tissue (**** = *p < 0.0001*). The same behaviour was identified for Protocol 3, but only for Amide I (** = *p < 0.01*). The results for each spectral absorption area were compared to the respective control with the Kruskal-Wallis test, followed by Dunn’s test.

FT-IR characterization is a valuable technique employed to thoroughly analyze and identify the collagen structures as the most dominant protein present within decellularized porcine uterine tissues. The mean FT-IR spectra (*n = 3*) for porcine native uterine tissue and decellularized tissues with different techniques are shown in Figures 7, 8. The following spectral regions for each sample can be categorized to: 1,480–1700 cm^-1^ for protein regions and 2,700–3,700 cm^−1^ for lipids. Sharp peaks at around 1,630, 1,538 and 1,234 cm^−1^ represent Amides I, II and III bands, respectively. Absorption bands around 1,449, 1,394, 1,336, 1,278, 1,234, 1,204 cm^-1^ attributed to the *δ*(CH_2_), *δ*(CH_3_), *ν*(C–N), and *δ*(N–H) absorption of collagens (Belbachir et al., 2009; Camacho et al., 2001). Native tissue and all decellularized groups exhibited absorptions at 1,080 and 1,032 cm^−1^ which attributed to *ν*(C–O) and *ν*(C–O–C) absorption of the collagen fibrils and carbohydrate moieties, respectively (Nashchekina et al., 2020; Bi et al., 2005). In all experiments, the Amide I peak was higher than Amide II which is representative of collagen fibers.

The absorption band positioned around 3,288 cm^−1^ is associated with N-H stretching of Amide A, and asymmetrical stretching bands of CH_2_ exhibited around 3,072 and 2,938 cm^−1^ indicate Amide B bindings (Veeruraj et al., 2015). Polypeptide backbone C–O stretching vibration for Amide I represents a hydrogen bond between the N-H stretch (X position) and C=O (Gly). For Amide II and III, the spectra show the bending vibration of N-H which is coupled with C-N stretching vibration and C-O-H/C-C stretching, respectively. Peaks at 1,394 and 1,449 cm^−1^ correspond to the CH_2_ wagging for proline from amino acids and collagen and carbonate band ʋ_2_(CO_3_^2−^), respectively. Peak vibration around 1,080 cm^−1^ corresponds to the C-O stretching mode of C-OH groups of essential amino acids like serine, threonine and tyrosine of collagen protein (Barnas et al., 2020). Overall, FTIR results represent the characteristic amino acids and intact collagen molecules in decellularized tissues.

### 3.4 Thermogravimetric characterization of dUECM

**FIGURE 9:**
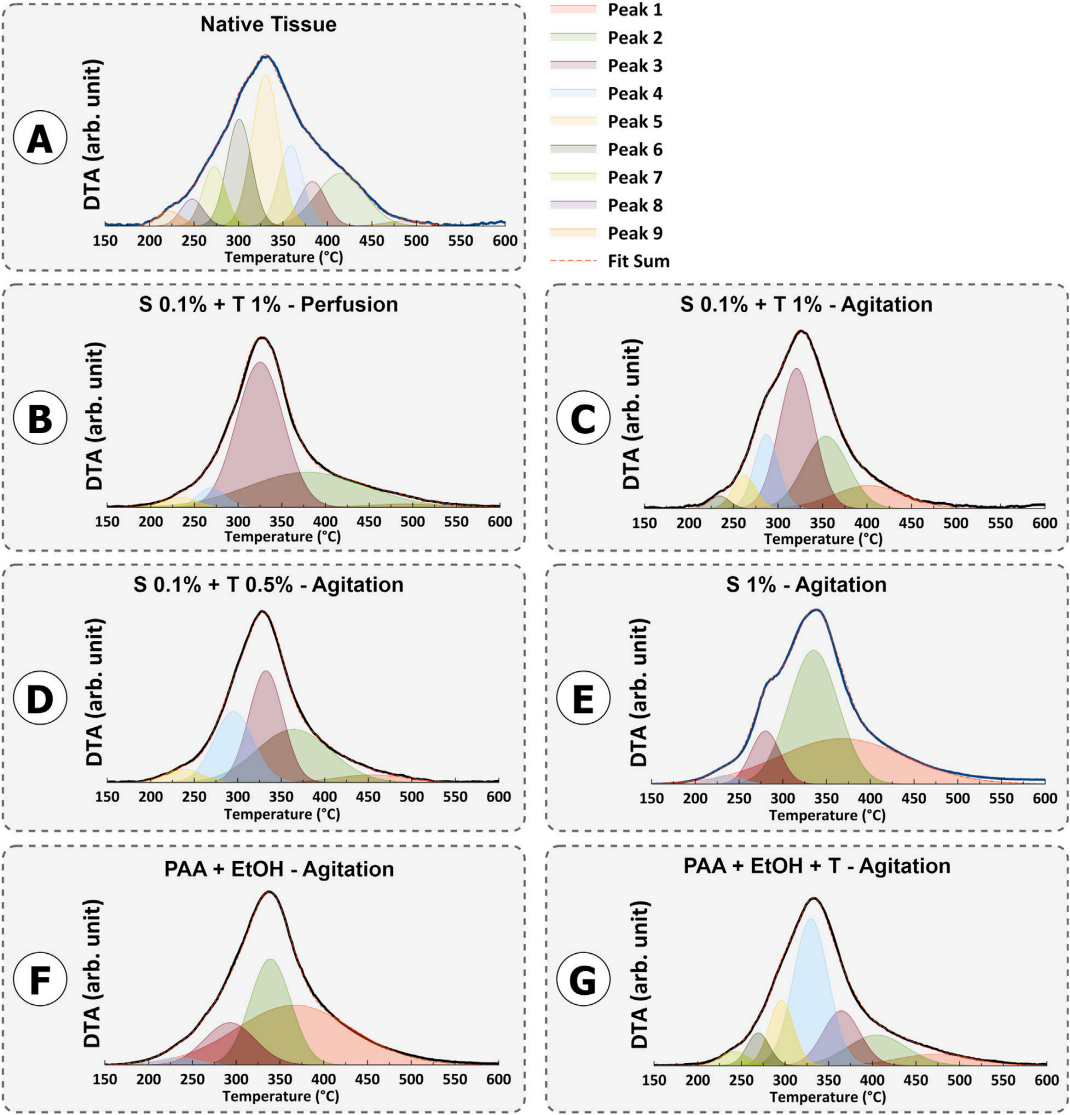
TG-DTA peaks of porcine uterine tissue **(A)** and dUECM samples for protocols 1-6 **(B–G)**.

The mass loss study for protein decomposition in porcine uterine tissue and decellularized samples is presented in Figure 9. In all samples, a weight loss between approximately 50°C and 100°C was observed, which corresponds to the evaporation of physiosorbed water. The decomposition of all samples mainly occurred between ∼ 200°C and 500°C. This decomposition can be attributed to the breakdown of proteins and the dehydration, deamination and then final decomposition of amino acid residues within peptide chains. Previous studies have confirmed that collagen decomposition mainly initiates around 300°C and ends around 500°C (Durga et al., 2022; Iafisco et al., 2012; Si et al., 2018; Lohrasbi et al., 2020).

The thermal degradation of collagen occurs in two steps: a rapid degradation between 160°C and 240°C, followed by a slower degradation between 300°C and 500°C (Akkouch, 2012).

**FIGURE 10:**
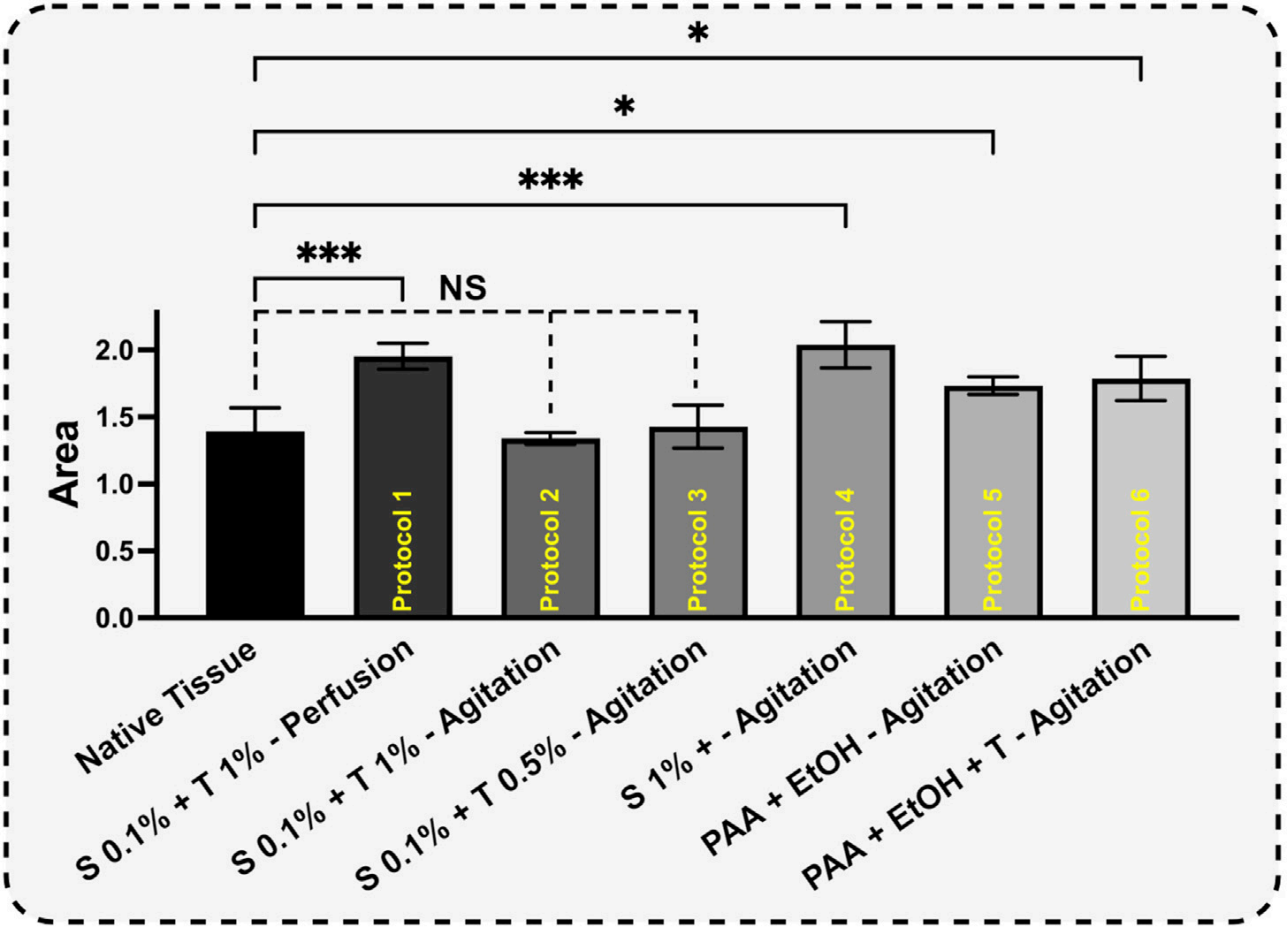
Based on curve-fitting results, the derivative decomposition peak areas were measured and compared. The surface area of each sample was measured to assess the extent of protein decomposition for each protocol (H). The results for each protocol were compared to the control using One-way ANOVA followed by the *post hoc* Dunnett test.

For Protocols 1 and 4, the remaining protein content was higher than that in the native tissue. This behavior indicates that, for a constant sample weight of 5 mg, one type of protein was more abundant than the other types of proteins. Removing or washing collagen type I from tissues is typically more demanding when compared to other collagen types. This increased challenge is primarily attributed to the structural and functional distinctions in the stability of various collagen types. Collagen type I, which is the predominant type found in various connective tissues, possesses a densely packed triple-helix structure with strong cross-linking, rendering it more resistant to denaturation than other collagen variants (Amirrah et al., 2022). The highest decomposition temperature observed in all samples was around 325°C, which corresponds to collagen type I. As mentioned earlier, the perfusion technique involving SDS 0.1% and Triton^®^ X-100 1% and 192 h agitation with SDS 1% resulted in thorough removal of smaller proteins such as laminin, fibronectin, hyaluronic acid, proteoglycans, and less robust collagen types (*p < 0.001*). On the other hand, SDS 0.1% + Triton^®^ X-100 1% and SDS 0.1% + Triton^®^ X-100 0.5% in agitation techniques show non-significant change compared to native tissue and were able to preserve the essential proteins needed (Figure 10).

### 3.5 XPS analysis of the total atomic composition of dUECM

**FIGURE 11:**
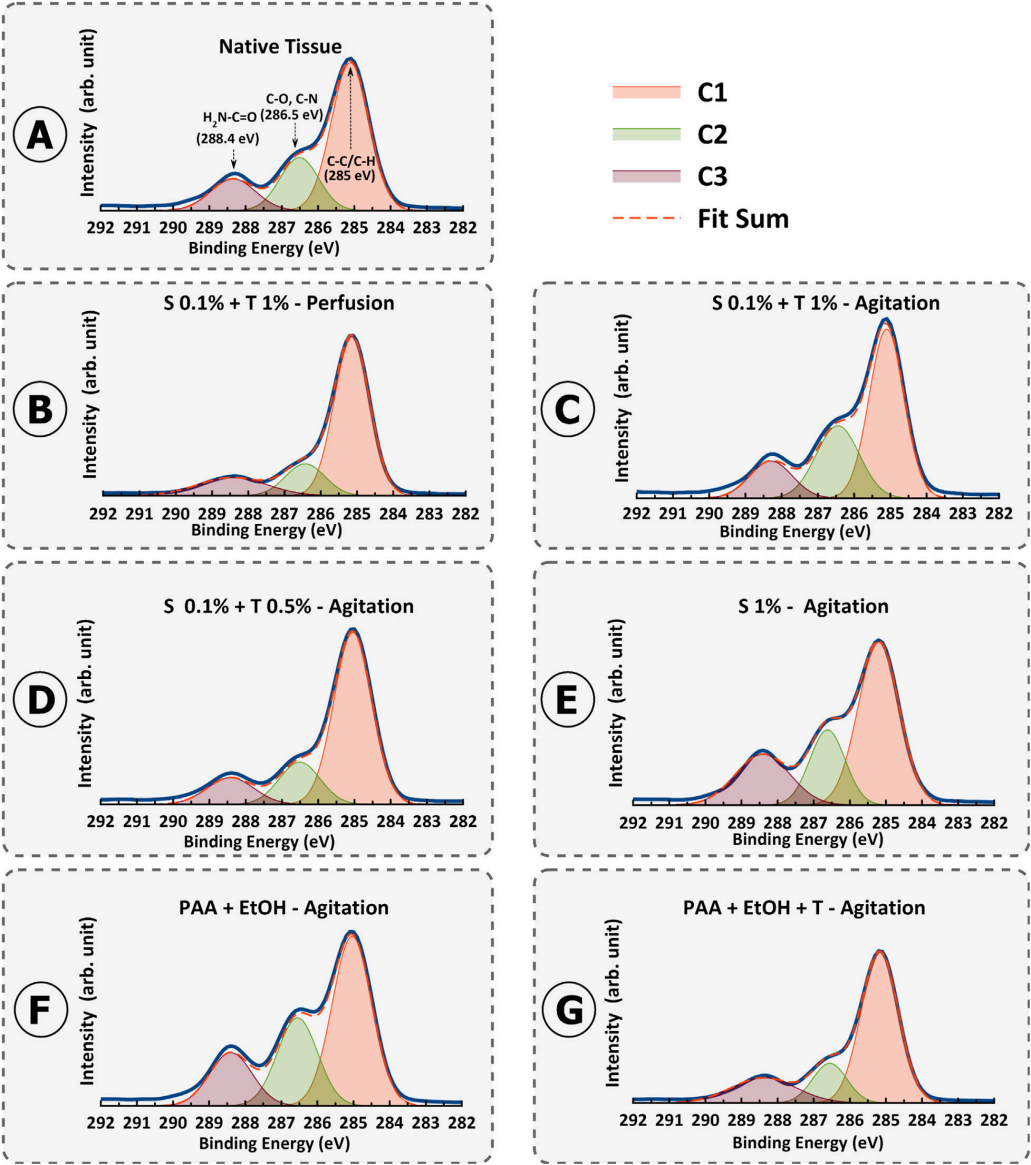
Fitted X-ray photoelectron spectroscopy (XPS) high-resolution region of C 1s spectrum for porcine uterine native tissue **(A)** and decellularization protocols 1-6 **(B–G)**.

XPS analysis was performed on the samples to investigate the atomic-level composition of the tissues. The results of the high-resolution XPS analysis can be found in Figure 11. This analysis specifically focused on phosphorus (P2p, 132 – 138 eV), carbon (C1s, 284 – 290 eV), nitrogen (N1s, 397 – 403 eV), and oxygen (O1s, 530 – 535 eV) levels to gain insights into tissue chemistry.

For carbon, we observed distinct peaks in the C1s spectra, which were subsequently deconvoluted into C1 (C-C/C-H), C2 (C-O/C-N), and C3 (H_2_N-C=O) components across all samples.

A specific focus was placed on the C3 peak FWHM values as an indicator of collagen preservation. A significant disparity in C3 FWHM values was observed among the protocols. Specifically, the agitation protocols (Protocols 2–6) demonstrated a lower FWHM for the C3 peak compared to the perfusion protocol (Protocol 1), suggesting potential variations in the presence or exposure of amino-carbonyl bonds associated with the H_2_N-C=O group (Figures 11B–G). These differences hold implications for the preservation of bioactive proteins and their structural properties during the decellularization process.

The lower C3 FWHM in the agitation protocols implies a better preservation of these amino-carbonyl bonds. This outcome resonates with collagen and distinct structural attributes and its susceptibility to changes induced by various decellularization protocols. Notably, the lower FWHM values of the agitation methods suggest that it might create a more conducive environment for retaining collagens by minimizing structural disruption.

Supplementary Table S1 provides a comprehensive insight into the XPS results, with a focus on the FWHM values and their connection to collagen (C3) alterations resulting from various decellularization methods. Examining the FWHM values alongside the C1/C3 ratios reveals a discernible pattern that reflects changes in the distribution of carbon-carbon (C-C/C-H) bonds and their potential implications for collagens preservation. Moreover, the C1/C3 FWHM ratios further strengthen the interpretation of collagen denaturation (lower ratio value means more denaturation) (Supplementary Table S1). For native uterine tissue, the C1/C3 FWHM ratio as the reference was 0.83. For Protocol 1 (0.1% SDS + 1% Triton^®^ X-100 – Perfusion), the C1/C3 FWHM ratio was calculated as 0.51, whereas for Protocol 2 (0.1% SDS + 1% Triton^®^ X-100 – Agitation), it was 0.75. For Protocol 3 (0.1% SDS + 0.5% Triton^®^ X-100 – Agitation), the ratio was 0.61. In protocol 4 (1% SDS – Agitation), the ratio decreased to 0.58, indicating a reduction in collagens compared to Protocols 2 and 3. Protocol 5 and 6 (PAA + EtOH ± Triton^®^ X-100 – Agitation) demonstrated the C1/C3 FWHM ratio of 0.8 and 0.79, respectively. These values align with expectations based on protein denaturation based on decellularization techniques. Broader C3 peaks and narrower C1 peaks, reflected in the higher C1/C3 FWHM ratios observed in protocols 5, 6, 2, 3, 4, and 1, respectively. These patterns in FWHM alterations correspond with the preservation or elimination of collagen, as noted in the various decellularization methods. This inconsistency supports the idea that more collagen is retained in the mentioned order of protocols, evident in their higher calculated values.

**FIGURE 12:**
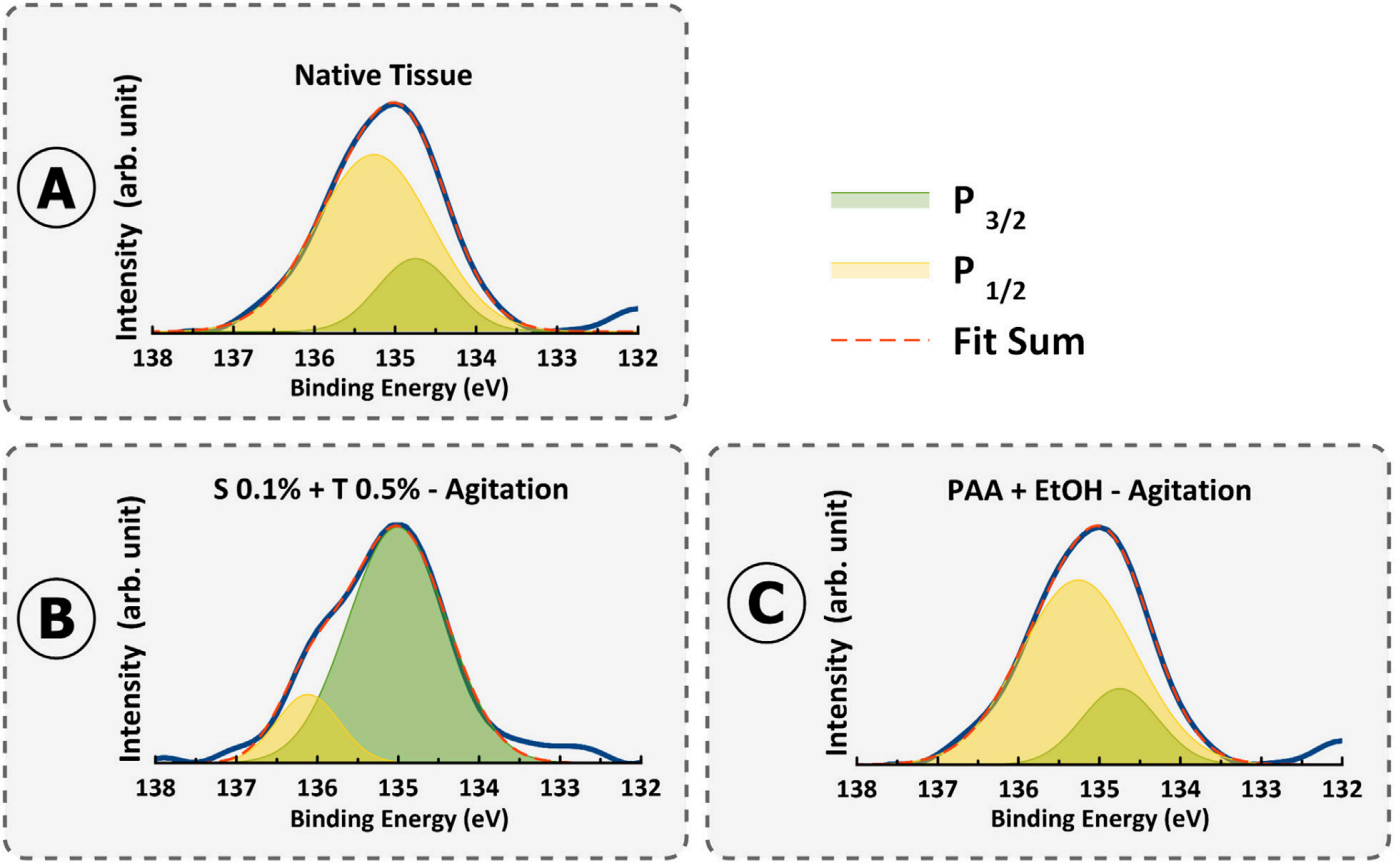
XPS spectra of P2p high resolution in **(A)** Native tissue, **(B)** S 0.1% + T 0.5% - Agitation, and **(C)** PAA + EtOH–Agitation.

Analysis of phosphorus atomic % provides information on DNA and cellular remnants (Figure 12). P2p spectra is the evidence of phosphates in phospholipids in cell nuclei (135.8 eV) (Lorusso et al., 1997). Remarkably, the trends observed across the methods reveal distinct patterns of DNA removal. Notably, treatments such as Protocol 5 (PAA + EtOH) and Protocol 3 (0.1% SDS + 0.5% Triton^®^ X-100) exhibit low atomic % values of phosphorus (0.12% and 0.4%), indicative of less effective DNA removal. The XPS results further validated the existence of DNA debris in these two specific protocols, while notably, phosphate was not detectable in the XPS results for the remaining protocols. This finding indicates that the XPS technique may not be a reliable method for quantifying DNA content in decellularized tissue. Instead, complementary assays such as DNA quantification, gel electrophoresis, and DAPI staining are essential to validate the effectiveness of decellularization.

## 4 Conclusion

Decellularized uterine extracellular matrix has emerged as a promising resource for uterine tissue engineering and regenerative medicine. In this study, we examined various protocols for decellularizing porcine uterine tissues and then characterized the outcomes in terms of DNA removal, bioactive-molecule preservation, and microstructural alterations.

Our results demonstrating that all protocols achieved decellularization with DNA removal, bioactive-molecule preservation, and microstructural alterations in the decellularized matrix depending on the protocols used. This highlights the importance of combinations of physical, chemical, and enzymatic components in decellularization for optimized outcomes.

Among the protocols examined, Protocol 2 (0.1% SDS + 1% Triton^®^ X-100 - Agitation) demonstrated its outstanding performance. DNase I and Triton^®^ X-100 are of pivotal factors in this protocol, playing their significant roles in optimizing the decellularization process. DNase I’s mechanism involves the specific cleavage of DNA into smaller fragments, facilitating effective removal of residual genetic material and reducing potential immunogenicity. Triton^®^ X-100 acts by disrupting cell membranes and solubilizing lipids and proteins, allowing for thorough removal of cellular debris while preserving the integrity of the ECM.

The preservation of GAGs and other vital bioactive molecules was notably superior in this protocol, highlighting the importance of the DNase I post-treatment in maintaining optimal levels of these components. This dual approach not only enhances the thoroughness of decellularization but also preserves the structural and functional integrity of key ECM components, such as collagens and GAGs.

Overall, this study contributes to the optimization of decellularization protocols for porcine uterine tissues and underscores the importance of careful protocol selection for the intended applications in tissue engineering. The combination of DNase I and Triton^®^ X-100 within Protocol 2 provides a valuable framework for achieving effective decellularization while preserving essential bioactive molecules, thus improving the potential for successful tissue engineering and regenerative medicine applications.

To overcome the limitations of this study, we would recommend more tests to be conducted in the future, along with the protocol optimization in terms of the test results or outcomes. These tests would include collagen quantification, proteomic analysis, immunogenicity, biocompatibility, residual chemical analysis, and mechanical testing on decellularized tissues, while the accordingly-optimized protocols would greatly facilitate the use of dUECM in the coming animal or human applications.

## Data Availability

The raw data supporting the conclusions of this article will be made available by the authors, without undue reservation.
