# Supplementary material for: Comparative analysis of porcine-uterine decellularization for bioactive-molecule preservation and DNA removal
Source: Front Bioeng Biotechnol. 2024 Oct 2;12:1418034. doi: 10.3389/fbioe.2024.1418034 (PMC11480021; doi:10.3389/fbioe.2024.1418034)
Supplement: Supplementary file 1 [file Table1.DOCX]

**Table S1:** Full Width at Half Maximum (FWHM) and quantitative evaluations for P-2p, O-1s, N-1s and C-1s regions for native tissue and decellularized samples

|  |  | **P 2p** | |  | **O 1s** | |  | **N 1s** | | |  | **C 1s** | | |  |  | |
| --- | --- | --- | --- | --- | --- | --- | --- | --- | --- | --- | --- | --- | --- | --- | --- | --- | --- |
| **Group** |  | **P 3/2** | **P 1/2** |  | **O1** | **O2** |  | **N1**  **C-NH_3_** | **N2**  **C-NH_2_** | **N3**  **N-O** |  | **C1**  **CC/C-H** | **C2**  **C-O, C-N** | **C3**  **H_2_N-C=O** | **C1/C2** | | **C1/C3** |
|  | Position | **134.7** | **135.4** |  | **531.7** | **532.8-534** |  | **399** | **400.5** | **401.5** |  | **285** | **286.5** | **288.4** |  | |  |
| **Native Tissue** | At. % | 0.27% | |  | 15.82% | |  | 8.82% | | |  | 75.1% | | |  | |  |
|  | Area | 41.54 | 191.81 |  | 17919.45 | 10136.30 |  | 9622.18 | 386.20 | 307.98 |  | 27673.51 | 9817.41 | 7235.75 | 2.81 | | 3.82 |
|  | FWHM | 1.11 | 1.68 |  | 1.65 | 1.96 |  | 1.42 | 0.93 | 0.97 |  | 1.24 | 1.23 | 1.50 | 1.01 | | 0.83 |
| **Protocol 1** | At. % | Not detected | |  | 13.09% | |  | 4.05% | | |  | 82.86% | | |  | |  |
|  | Area | 0 | 0 |  | 14456.38 | 3695.38 |  | 4548.92 | 327.49 | 25.72 |  | 26450.64 | 5947.03 | 5996.18 | 4.44 | | 4.41 |
|  | FWHM | 0 | 0 |  | 1.69 | 1.63 |  | 1.45 | 1.77 | 0.61 |  | 1.15 | 1.28 | 2.26 | 0.90 | | 0.51 |
| **Protocol 2** | At. % | Not detected | |  | 18.14% | |  | 10.87% | | |  | 70.99% | | |  | |  |
|  | Area | 0 | 0 |  | 24649.99 | 5591.59 |  | 11137.97 | 386.62 | 139.17 |  | 22618.42 | 8974.76 | 9353.30 | 2.5 | | 2.41 |
|  | FWHM | 0 | 0 |  | 1.64 | 1.88 |  | 1.43 | 1.07 | 0.93 |  | 1.34 | 1.16 | 1.78 | 1.16 | | 0.75 |
| **Protocol 3** | At. % | 0.4% | |  | 15.07% | |  | 8.32% | | |  | 76.21% | | |  | |  |
|  | Area | 223.79 | 41.47 |  | 9366.27 | 16406.04 |  | 8479.95 | 727.12 | 136.22 |  | 23745.18 | 12554.29 | 6346.94 | 1.89 | | 3.74 |
|  | FWHM | 1.44 | 0.92 |  | 1.36 | 1.92 |  | 1.34 | 1.76 | 0.92 |  | 1.20 | 1.19 | 1.96 | 1.01 | | 0.61 |
| **Protocol 4** | At. % | Not detected | |  | 15.63% | |  | 6.54% | | |  | 77.83% | | |  | |  |
|  | Area | 0 | 0 |  | 18078.74 | 12427.05 |  | 7960.00 | 587.68 | 189.79 |  | 31855.87 | 8950.26 | 6861.41 | 3.55 | | 4.64 |
|  | FWHM | 0 | 0 |  | 1.54 | 2.20 |  | 1.35 | 1.47 | 1.01 |  | 1.22 | 1.12 | 2.11 | 1.09 | | 0.58 |
| **Protocol 5** | At. % | 0.12% | |  | 18.73% | |  | 5.78% | | |  | 75.37% | | |  | |  |
|  | Area | 39.37 | 145.23 |  | 29368.58 | 1736.80 |  | 6608.58 | 355.30 | 163.55 |  | 28445.34 | 7490.04 | 5435.25 | 3.79 | | 5.23 |
|  | FWHM | 1.11 | 1.68 |  | 1.66 | 1.17 |  | 1.39 | 1.34 | 1.13 |  | 1.23 | 1.32 | 1.52 | 0.93 | | 0.80 |
| **Protocol 6** | At. % | Not detected | |  | 20.77% | |  | 9.86% | | |  | 69.38% | | |  | |  |
|  | Area | 0 | 0 |  | 36719.59 | 2910.68 |  | 11275.79 | 540.86 | 151.39 |  | 22606.85 | 11944.54 | 7656.67 | 1.89 | | 2.95 |
|  | FWHM | 0 | 0 |  | 1.71 | 1.58 |  | 1.39 | 1.19 | 0.79 |  | 1.35 | 1.20 | 1.71 | 1.12 | | 0.79 |
